# Climate‐induced increase in terrestrial carbon storage in the Yangtze River Economic Belt

**DOI:** 10.1002/ece3.7414

**Published:** 2021-06-04

**Authors:** Fengxue Gu, Yuandong Zhang, Mei Huang, Li Yu, Huimin Yan, Rui Guo, Li Zhang, Xiuli Zhong, Changrong Yan

**Affiliations:** ^1^ Key Laboratory of Dryland Agriculture, Institute of Environment and Sustainable Development in Agriculture Chinese Academy of Agricultural Sciences Beijing China; ^2^ Key Laboratory of Forest Ecology and Environment of National Forestry and Grassland Administration Research Institute of Forest Ecology, Environment and Protection Chinese Academy of Forestry Beijing China; ^3^ Key Laboratory of Ecosystem Network Observation and Modeling Institute of Geographical Sciences and Natural Resources Research Chinese Academy of Sciences Beijing China; ^4^ National Climate Center China Meteorological Administration Beijing China

**Keywords:** carbon storage, climate change, process‐based model, Yangtze River Economic Belt

## Abstract

Predicting the change in carbon storage in regions of high carbon uptake and those under highly intensive human disturbance is crucial for regional ecosystem management to promote sustainable development of the economy and ecology in the future. We use a process‐based model to estimate the terrestrial carbon storage in Yangtze River Economic Belt (YREB) and to predict the change of carbon storage over the next 100 years. The results show that the vegetation carbon (VC) and soil organic carbon (SOC) storage were 8.97 and 28.85 Pg C in the YREB from 1981 to 2005, respectively. The highest VC density is distributed in the southern region of the YREB, and the highest SOC density distributes in subalpine and alpine area of the western region of the YREB. Carbon storage in the YREB continued to increase from 1981 to 2005 and in future projections, under both the representative concentration pathway 4.5 (RCP4.5) and the RCP8.5 scenarios. The increased rate of carbon storage in the YREB under the RCP8.5 scenario is higher than that under the RCP4.5 scenario. Under the RCP4.5 scenario, the increasing trend of VC storage tends to be reduced after the 2060s; conversely, the increase of both VC and SOC is accelerated after the 2050s under the RCP8.5 scenario. The SOC density in Western Sichuan will decrease in the future, especially under the RCP8.5 scenario. Western Sichuan has the highest SOC density in the YREB; therefore, it is important to manage the ecosystems there in order to cope with significant warming. The positive impact of warming and the CO_2_ fertilization effect on vegetation growth and carbon uptake will be predominantly attributed to the increase of terrestrial carbon storage in the YREB. However, warming will stimulate the decomposition of soil organic carbon, contributing directly to reducing SOC storage in high‐altitude regions (e.g., alpine and subalpine regions of Western Sichuan).

## Graphical abstract

The vegetation and soil carbon storage is 8.97 Pg C and 28.85 Pg C, respectively, which accounts 42.13% and 26.04% of Chinese terrestrial ecosystems. Rising temperature and elevated atmospheric CO_2_ concentration stimulate the increase of carbon storage in Yangtze River Economic Belt, and the increase is higher under RCP8.5 than that under RCP4.5. Soil carbon storage in western Sichuan province, where has the highest soil carbon storage, decrease in the future under both scenarios.

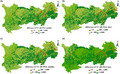

## 1 INTRODUCTION

Carbon sequestration in soil and vegetation is the most important means of reducing atmospheric CO_2_ concentration (Lenton, 2014). Accurate estimation of terrestrial carbon storage in unique regions is important for understanding the role of terrestrial ecosystem as source or sink of atmospheric CO_2_ (Ni, 2013). Magnitude and dynamics of terrestrial carbon pools are also indicators of ecosystem health because they represent the comprehensive outcomes of various processes of ecosystems (Lal, 2003). A large number of efforts have been carried out attempting to estimate the global or regional carbon storage of ecosystems and to determine the change of carbon storage due to global changes (Cao et al., 2003; Felzer et al., 2004, 2005; Melillo et al., 1993), but those estimations were reported with large uncertainties. The estimation of global soil organic carbon (SOC) varies from 1,000 to 2,200 Pg C (Bruce et al., 1999; Eswaran et al., 1993; FAO, 2015; Lal, 2004, 2008; Lenton & Huntingford, 2003; Post et al., 1982; Stockmann et al., 2013). The vegetation carbon (VC) pool was estimated to be 268–901 Pg C (Lal, 2004, 2008; Lenton & Huntingford, 2003). The estimations reported for VC and SOC storage in China also covered large ranges: 6.1–76.2 Pg C for VC storage and 43.6–185.7 Pg C for SOC storage (Lal, 2002; Ni, 2013; Xu et al., 2018; Yu et al., 2010). Significant differences in estimation of terrestrial carbon storage are mainly attributed to methods, scale, data availability, etc. (Ni, 2013; Stockmann et al., 2015). In addition, most efforts have provided the magnitude of carbon stock at a particular time but did not describe the temporal trend, especially on large scales (Stockmann et al., 2015). Ecosystem models provide a useful method to assess the magnitude and dynamic of carbon pools, and to predict the future terrestrial carbon storage change in response to climate change (Cao & Woodward, 1998a; Melillo et al., 1993; Peng et al., 2014; Ren et al., 2012; Sitch et al., 2003; Tian et al., 2011).

Small shifts in carbon stored in soil and vegetation can result in a large change of atmosphere CO_2_ concentration and climate (Lal, 2004; Pregitzer & Euskirchen, 2004). The dynamics of the terrestrial carbon storage are influenced by multiple global change drivers, such as elevated CO_2_ concentration, climate change, nitrogen deposition, and phosphorus addition (Luo et al., 2011; Yue et al., 2017), land use and land cover change, human disturbance, etc. (Brovkin et al., 2013; Luo et al., 2017; Pregitzer & Euskirchen, 2004). Climate change would result in the redistribution of vegetation type and a change in productivity, which then would bring about changes in biomass and soil carbon content (Adams et al., 1990; Lal, 2004; Post et al., 1982; Prentice & Fung, 1990; Trumbore, 1997). Elevated atmospheric CO_2_ concentration has been reported to have stimulated plant photosynthetic capacity and, consequently, increase the biomass and soil carbon storage (Prentice et al., 2011). Various results suggest the positive response of carbon storage to elevated CO_2_ concentration and a negative response to climate change (Cox et al., 2013; Friedlingstein et al., 2006; Mao et al., 2009; Peng et al., 2014; Zickfeld et al., 2011); however, all of these drivers also contribute uncertainty about the stability of carbon pools (Arora et al., 2013; Sitch et al., 2008), as the drivers have both positive and negative feedback on ecosystems (Pendall et al., 2011). Although climate change is considered as one of the dominant drivers to influence the carbon sequestration in ecosystems (Bjorkman et al., 2018; Nemani et al., 2003), the effects are likely to vary in different regions, and to date, its regional pattern is poorly understood (Arneth et al., 2010).

Benefited from the East Asian monsoon, the mid‐ to low‐latitude region in eastern China contains distinctive subtropical forests with high CO_2_ uptakes, which have been illustrated by long‐term eddy covariance observations (Yu et al., 2014). Yangtze River Economic Belt (YREB), including 11 provinces and cities, is the most important ecological economic region in this area, and it not only has the largest area of subtropical forests, but also the second largest natural forests in China, which are distributed in alpine and subalpine areas of the western region of the YREB. The forest area accounts for 41.04% of the total forest area in China, although the land area only accounts 21.9% of the total land area of China. At the same time, this area has experienced a long history of agricultural exploitation and land use change and is one of the most important major grain producing areas in China. Thus, the terrestrial ecosystems of the YREB play an important role in the carbon budget of China. However, an estimate of the terrestrial ecosystem storage and its response to future climate change is not yet available. As a major national strategic developed region, the YREB leads in the construction of ecological civilization in China, and it will provide a general model of a high‐efficiency economy and a better environment for other inland river economic zones. Assessing the spatial–temporal variations of carbon storage in this region is vital for evaluating ecosystem quality and strengthening the ability and strategy to respond in the face of future climate change (Stockmann et al., 2015). In addition, as one of the major grain producing areas of China, the evaluation of carbon storage and its trend under future climate change is also important for food security, because SOC can improve the water and nutrient holding capacity and supply, soil structure, and biotic activity and the crop yield is stimulated by an increase of SOC storage (Lal, 2004).

The 5th Assessment Report of the Intergovernmental Panel on Climate Change (IPCC, 2014) indicated that the increase of global mean surface temperature by the end of 21st century (2081–2,100) relative to 1986–2005 is likely to be −0.3–4.8°C under different projections of greenhouse gas emission. Moreover, the simulation from the Coupled Model Intercomparison Project 5 (CMIP5) models showed that the increase of mean surface temperature over China and Asia would be higher than the increase of global mean surface temperature (Shao et al., 2013). It is critical then to understand the following: (a) In what ways the carbon stock of terrestrial ecosystems will respond to future global warming; and (b) the relationships between rising temperature and elevated atmospheric CO_2_ concentration and storage of carbon in regional ecosystems. It is vitally important to understand these points for the YREB with its diverse climate, landform, and vegetation types. In this study, the spatial patterns of VC and SOC density change responding to past and future climate change in the YREB were investigated, based on a process‐based ecosystem model, which has been used to simulate the global/regional terrestrial carbon cycle and its response to global climate change (Cao & Woodward, 1998a, 1998b; Gu, Zhang, Huang, Tao, Guo, et al., 2017; Tao et al., 2007), providing a reliable and acceptable estimation of productivity and carbon storage in China (Gao et al., 2012; Yu et al., 2010). The analysis gives an insight into the potential change of carbon storage through the 21st century affected by climate change and CO_2_ fertilization under medium and high CO_2_ emission scenarios based on the 5th IPCC’s Assessment Report (IPCC, 2014).

## 2 MATERIALS AND METHODS

### 2.1 Study area

**FIGURE 1:**
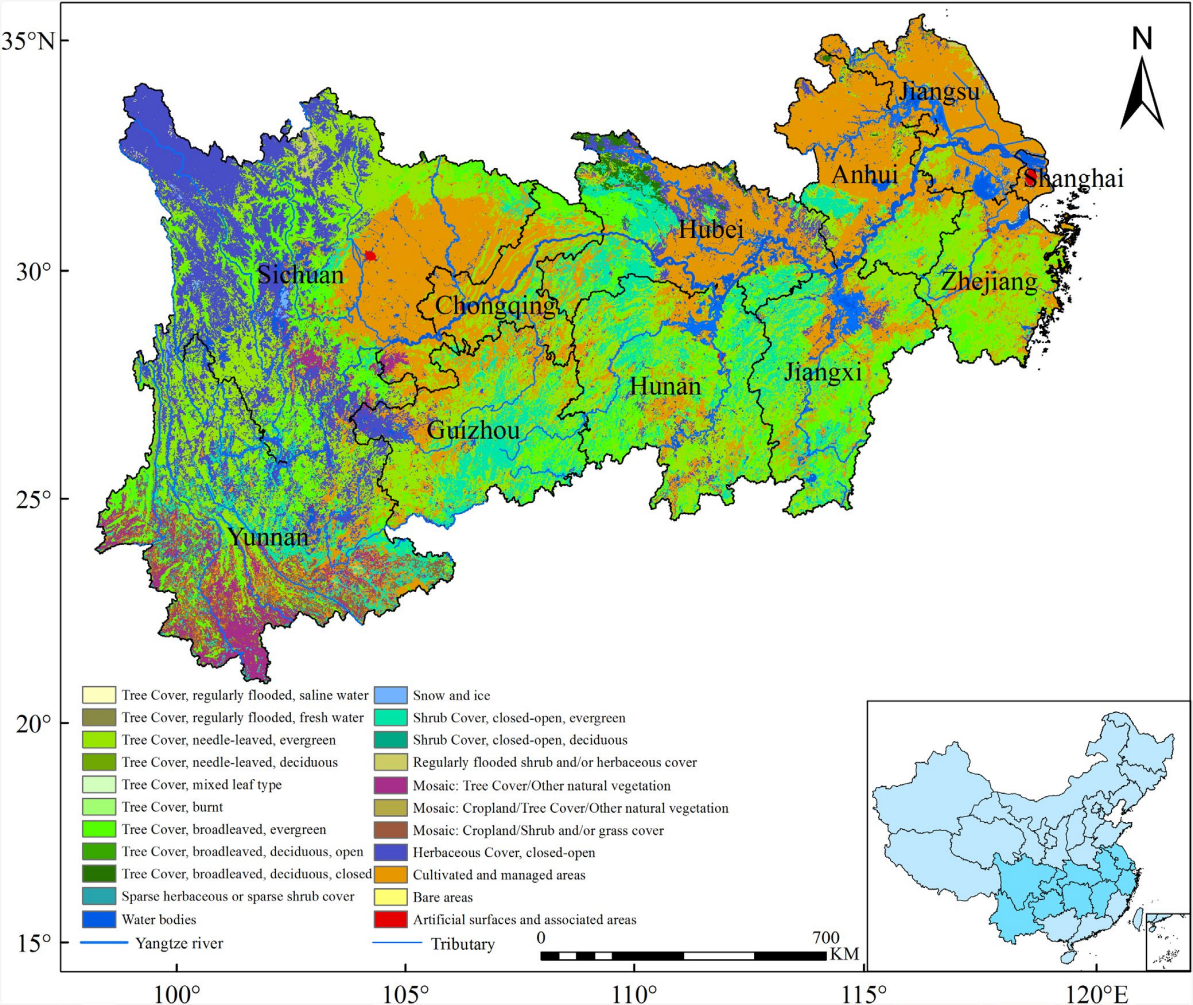
Location of the YREB and the land use and land cover (data from GLC2000 (Bartholomé & Belward, 2005))

The YREB is located in the middle China, within latitudes of 90°31′50″‐121°53′23″E and longitudes of 21°8′45″–34°56′47″N. The total area is roughly 2.05 million km^2^. Most areas are typical of a subtropical monsoon climate. The area has diverse topography, soil types, land use, and land cover (Figure 1). The distribution of soil types has distinctive regional characteristics. Soil types mainly include Haplic Acrisol and Haplic Luvisol, as the main soils classes (FAO classification). Forest area is 70.35 million ha and is the largest vegetation type in area in the YREB. The forest coverage is 40.69% and accounts for 41.04% of the total Chinese forest (http://www.stats.gov.cn/).

### 2.2 Model description

The CEVSA2 (Carbon Exchange between Vegetation, Soil, and the Atmosphere) model is a process‐based ecosystem model that simulates energy transfer, carbon, nitrogen, and water cycles in the vegetation–soil–atmosphere system. It is designed to quantify the responses of ecosystem processes to global changes such as atmospheric CO_2_, climate, and nitrogen deposition (Cao & Woodward, 1998a, 1998b; Gu et al., 2015). The CEVSA2 model has extensive applications to evaluate the impacts of climate change on terrestrial ecosystems and to assess the vulnerability of terrestrial ecosystems to climate change on a national scale (Cao et al., 2003; Gu, Zhang, Huang, Tao, Guo, et al., 2017; Gu, Zhang, Huang, Tao, Li, et al., 2017; Gu et al., 2015; Tao et al., 2003, 2007; Yu et al., 2006) and different regional scales (Gu et al., 2014; Pang et al., 2012; Zhang et al., 2013, 2016). In the CEVSA2 model, SOC is the dynamic of carbon input by photosynthetic production and carbon loss by soil heterotrophic respiration. It is calculated as follows:

(1) $$\frac{dSOC_{i}}{\mathrm{dt}}=SOC_{i‐1}+\left(LT‐HR\right)$$

where *SOC*_i_ is SOC density, and *SOC*_i‐1_ is existing SOC density. *LT* is the carbon input as litter into soils. In the CEVSA2 model, carbon allocated to plant organs, including leaves, stems, and roots, will be lost into soils based on a given mean residence time with a statistical distribution (Lloyd & Farquhar, 1996). The seasonality of litter production is estimated based on the method described by Box (1998). *HR* is the loss of carbon by SOC decomposition. The decomposition of litter and soil organic matter into CO_2_ is simulated based on the CENTURY model (Cao & Woodward, 1998b; Parton et al., 1987, 1988, 1993). Finally, *i* is the time step of simulation.

VC storage is the accumulation of biomass subtracting loss of carbon by litter fall, and it is calculated as follows:

(2) $$\frac{dVC_{i}}{\mathrm{dt}}=VC_{i‐1}+\left(A_{i}‐AR_{i}‐LT_{i}\right)$$

where *VC*_i‐1_ is existing biomass. *A*_i_ is the gross carbon by assimilation, and *AR*_i_ is the total plant autotrophic respiration.

### 2.3 Data sources

The meteorological data driving the CEVSA2 model include mean air temperature, precipitation, relative humidity, and cloud cover at a 10‐day time step. The dataset for 1961 to 2099 was simulated by BCC_CSM1.1, which is the latest version of regional climatic system model developed by National Climate Center of China. The simulations from 1961 to 2005 were calibrated by observations from national reference climatological stations. Then, the dataset for 2006 to 2099 was simulated by BCC‐CSM1.1 which was driven by radiation forcing of the two representative concentration pathways (the RCPs) scenarios. The RCP4.5 scenario assumes that global annual greenhouse gas emission will peak around 2040, then decline. In the RCP8.5 scenario, emissions continue to rise throughout the 21st century (van Vuuren et al., 2011). All the RCP scenarios describe greenhouse gas concentration trajectories and provide an important approach to assess future climate change and its effects for projections of the future (IPCC, 2013; van Vuuren et al., 2011). All of these climate outputs were downscaled to a 0.1° latitude /longitude data grid by using ANUSPLIN software (Hutchinson, 1989). Annual atmospheric CO_2_ concentrations in historical and future projections were downloaded from the Data Distribution Centre of IPCC (http://www.ipcc‐data.org/). To estimate the impact of different future climate change projections, we used results of the slice of 1986–2005 as a reference period suggested by the 5th IPCC’s Assessment Report (IPCC, 2014). The land cover data were resampled from 1km resolution Global Land Cover 2000 database (Bartholomé & Belward, 2005). The dataset included 22 land cover types, and the 0.1° × 0.1° spatial simulation unit has a unique and fixed land cover type. The soil data and parameters were derived from the digitalized 1:14,000,000 soil texture map of China (The Institute of Soil Science & CAS, 1986).

To validate the simulations of the CEVSA2 model, we collected published VC and SOC density datasets from references (Tang et al., 2018; Wang et al., 2000). Wang et al. (2000) provided 2,473 and 784 SOC density data measurements for China and the YREB, respectively. The average depth of the soil profiles used by Wang et al. (2000) was 87cm, and the observations were conducted between 1978 and 1985. Tang et al., (2018) provided 14,371 SOC and VC density data measurements in China, of which 5,372 data points were observed in the YREB, with the depth of the soil profiles being 1 m. The observations of Tang et al. (2018) were conducted between 2011 and 2015. All these profile data included in observations were from all types of vegetation and soil. In the 0.1° simulation unit, we calculated the average value of samples with the same vegetation type to validate the simulation. The CEVSA2 model calculated the SOC density in the entire soil depth. Both the SOC density datasets obtained from different resources have different soil profile depths; we compared the simulations and observations directly because any conversion might bring extra errors into the observations.

### 2.4 Model simulations and validation

To obtain the initial state parameters of the CEVSA2 model simulation, the CEVSA2 model was firstly spun until it reached equilibrium status driven by the 30‐year averaged climatic data (1971‒2000) and a fixed CO_2_ concentration level based on the 30 years mean value. Then, the simulation was conducted using transient climate and atmospheric CO_2_ concentration data for the period of 1961 to 2099, which was called the “dynamic simulation.” The CEVSA2 model was run at a 0.1° × 0.1° spatial resolution in continental China with a 10‐day time step. To eliminate the impact of assumed initial state, the model was run repeatedly for dynamic simulation, and then, the modeling results from 1981 to 2099 were used to project the responses of terrestrial carbon storage in the YREB for this study.

**FIGURE 2:**
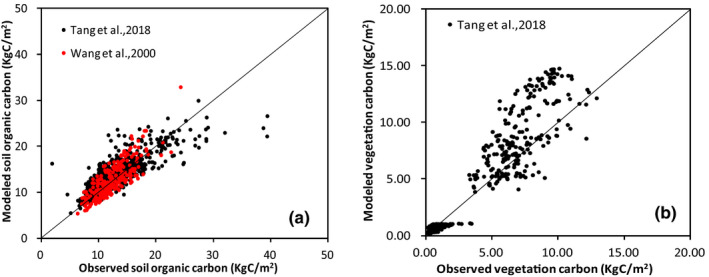
Comparison of simulated and observed SOC density (a) and VC density (b) in the YREB

During the application of the CEVSA2 model, a great deal of validation was carried out. However, previous work focused on the validation of the simulated net primary productivity (NPP) by using plot‐sampling observations (Cao & Woodward, 1998b; Gu, Zhang, Huang, Tao, Guo, et al., 2017) and gross primary productivity, ecosystem respiration, net ecosystem exchange, and evapotranspiration by using eddy flux tower data (Gu et al., 2006; Tao et al., 2007). Simulated SOC storage and VC storage by the CEVSA2 model for China are within the range of all observation and simulation‐based estimations (Ni, 2013; Yu et al., 2010). In addition, the simulated SOC storage is exceptionally close to the estimation made by Yu et al. (2005) which is based on a 1:1,000,000 soil database including 7,292 soil profiles. However, limited by the availability of observation data, there has been lack of validation of simulated SOC and VC density. The datasets from the two references (Tang et al., 2018; Wang et al., 2000) provided the observation data to validate the model simulation. The observations covered multiple time periods ranging from the 1980s to 2010s, and thus, the average value of SOC density during 1981‒2005 estimated by the CEVSA2 model was compared with the observations of Wang et al. (2000) and Tang et al. (2018). The simulations explained the spatial variations of observed SOC densities well (Figure 2a). Obviously, the CEVSA2 model overestimated VC density (Figure 2b). Besides inconsistency of spatial resolutions and errors in observations, two other reasons were considered as the sources of overestimation of VC density by the CEVSA2 model. Firstly, the CEVSA2 model does not consider the effects of all kinds of disturbance, such as harvesting, afforestation, deforestation, fire, and extreme climate events. Secondly, China has carried out programs to conserve and expand forest since 1980s, and forest cover continues to grow (http://www.stats.gov.cn/). China leads in greening of the world, and the increase in greening in China is mainly due to forest regrowth (Chen, Park, et al., 2019). Forests in the Yangtze River Basin have also increased significantly (Kong et al., 2018); therefore, there are more young and middle‐aged forests in this area. The simulation made by the CEVSA model is based on the equilibrium state hypothesis and based on the fact that forest ecosystems reach maturity. Although many studies have revealed that middle‐aged and near‐mature forest had higher productivity than other forests (Li, Fang, et al., 2015; Li Xu & Zhang, 2015), their accumulation of carbon in the ecosystem was still lower than that of mature forests.

In addition, there are also many previous studies which focused on the estimation of forest carbon storage in the YREB; however, they were limited to one administration cell or a special forest type in a small region, etc. The estimation had large spatial variations in previous studies, and VC density ranged from 603 g C m^‐2^ to 4,906 g C m^‐2^, and SOC density ranged from 9,051 g C m^‐2^ to 27,667 g C m^‐2^ (Cao & Li, 2012; Li, Fang, et al., 2015; Li et al., 2012, 2013, 2016; Li, Xu, et al., 2015; Liu et al., 2017; Xi et al., 2010). Although the model simulation has the differences from the observation from a special region and forest type, it is within the range of observations and describes the variations among regions as well.

### 2.5 Data processing

In this study, the period of 1981‒2005 represents the current state, as China has experienced great changes and entered a period of high development since the 1980s. The period of 1986‒2005 is defined as the reference period for the projected changes suggested by the 5th IPCC’s Assessment Report (IPCC, 2014), and the periods 2021‒2050 and 2070‒2099 are used to indicate the middle and late 21st century, respectively. We calculated the average values for all the different periods and the different scenarios, and then the difference between average values of middle, late 21st century, and the reference period, respectively.

## 3 RESULTS AND DISCUSSION

### 3.1 Terrestrial carbon storage in the YREB during 1981‒2005

The mean VC and SOC density and the regional total VC and SOC storage in the YREB are shown in Table 1. The terrestrial carbon storage in YREB was 37.82 Pg C on average during 1981‒2005, and SOC storage in the YREB is about three times that of VC storage. In the YREB, forest has the highest carbon density and accounts for 58.7% of the total regional carbon storage. Cropland is the second largest land cover in the YREB but has the lowest carbon density (Table 2). Shrubland has the lowest total carbon storage, which only accounts for 8.7% of the total regional terrestrial carbon storage.

**TABLE 1:** Average carbon density during 1981‒2005 and future under different scenarios

Period		Average carbon density (g C/m^2^)			Average carbon storage (Pg C)		
		Vegetation	Soil	Total	Vegetation	Soil	Total
Present	1981‒2005	4,558	14,657	19,215	8.97	28.85	37.82
	1986‒2005	4,574	14,665	19,239	9.00	28.87	37.87
The RCP4.5	2006‒2099	5,253	15,240	20,493	10.34	30.00	40.34
	2021‒2050	5,053	15,008	20,061	9.95	29.54	39.49
	2070‒2099	5,613	15,649	21,262	11.05	30.80	41.85
The RCP8.5	2006‒2099	5,575	15,396	20,971	10.97	30.30	41.27
	2021‒2050	5,117	15,031	20,148	10.07	29.59	39.66
	2070‒2099	6,382	16,036	22,418	12.56	31.56	44.12

**TABLE 2:** Average carbon density in different types of vegetation during past 25 years and in the future under different scenarios (unit: g C/m^2^)

Vegetation type	1981‒2005			2006‒2099 (under the RCP4.5 scenario)			2006‒2099 (under the RCP8.5 scenario)		
	Vegetation	Soil	Total	Vegetation	Soil	Total	vegetation	Soil	Total
Forest	10,446	17,357	27,803	11,322	17,580	28,902	11,989	17,640	29,629
Shrubland	818	13,480	14,298	964	14,337	15,301	1,048	14,625	15,673
Grassland	453	16,747	17,200	530	17,471	18,001	577	17,605	18,182
Cropland	825	12,396	13,221	967	13,122	14,089	1,048	13,389	14,437

The carbon stock of ecosystems in the YREB, especially forest ecosystems, plays an important role in the Chinese terrestrial carbon budget. The average VC and SOC density of Chinese terrestrial ecosystems during 1981‒2005 simulated by the CEVSA2 model was 2,263 and 12,036 g C/m^2^, respectively. It is obvious that the VC and SOC density in the YREB are higher than the mean value of Chinese terrestrial ecosystems. The VC storage is about 42.1% of all Chinese terrestrial ecosystems although the area only accounts for 21.4% of the whole country. The SOC storage accounts for 26.0% of Chinese terrestrial ecosystems. In the YREB, forest contributes 58.7% of total regional carbon stock. The vegetation, soil, and total carbon density of forests are higher than the mean values of China and world forests (Sun et al., 2016; Tang et al., 2018; Zhou et al., 2010). With the growth of forest cover and forest ecosystem management in the YREB (National Development & Reform Commission, 2020), the carbon storage in this region will play a more important role in Chinese carbon sequestration management. The Yangtze River Basin has been the most important agricultural production area of China since ancient times (Li, Xu, et al., 2015; Li Yang & Cao, 2015). The cropland area is about 20.8% of the total land area of the YREB, but only contributes 19.8% of the total carbon storage. However, the soil in the cropland ecosystems has experienced cultivation for a long period of time and depletion of the carbon stock occurred. This ecosystem has high potential to sequestrate carbon if a series of recommended management practices for agricultural ecosystems are practiced (Lal, 2002, 2008). Therefore, the cropland soil in the YREB will have high potential for sequestration of carbon in the future.

**FIGURE 3:**
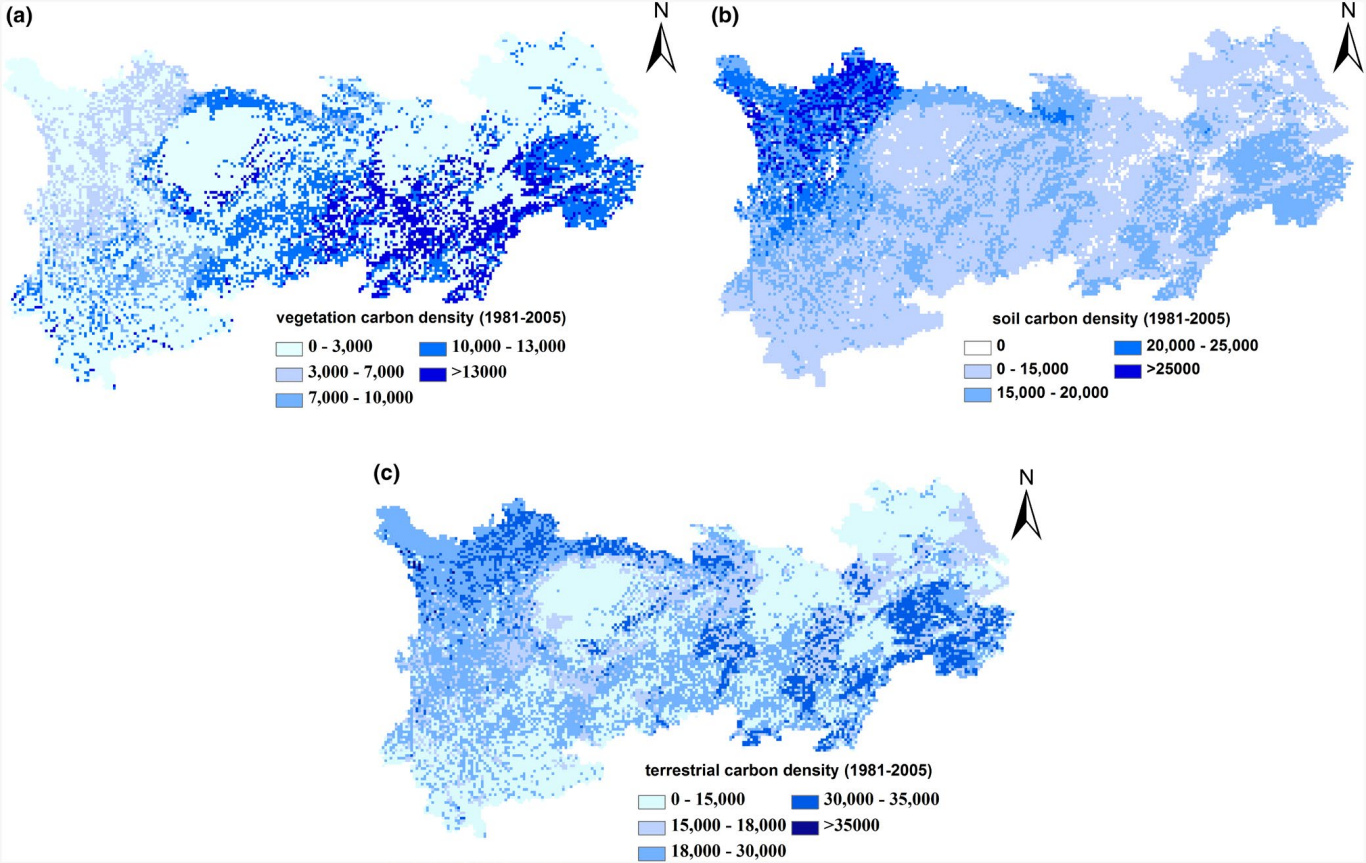
Spatial pattern of VC density (a), SOC density (b) and terrestrial carbon density (c) in the YREB during 1981‒2005. Unit: g C/m^2^

The land carbon density in the YREB shows large spatial variations. The highest VC density is distributed in mountainous areas of the southern YREB, including Zhejiang, Jiangxi, and Hunan province, mostly above 10,000 g C/m^2^ (Figure 3a). SOC densities in the subalpine and alpine soils in the western YREB suggest the highest value, mostly above 20,000 g C/m^2^ (Figure 3b). The lowest VC and SOC densities are distributed in plains of the northern YREB, including Jiangsu, Anhui, Hubei province, and the Chengdu Plain. Determined by spatial patterns of VC and SOC density, the western region of the Sichuan province, and surroundings of Sichuan Basin, Hunan, Jiangxi, and Zhejiang province have the highest carbon density (Figure 3c).

The spatial distribution of VC density is largely dependent on the NPP of ecosystems, which is decided by the distribution of forest in the YREB. The northeastern region of the YREB is dominated by farmland and has the lowest VC density. Forest coverage in the southern part of the YREB is higher than that in the northern, and a large area of subtropical forest is distributed throughout the southern YREB (Yu et al., 2014). According to the 8th national forest inventory, forest cover rates of Jiangxi and Zhejiang provinces are the second and third highest in China. In addition, all provinces in the YREB, especially Hunan, Yunnan, and Hubei province, located in the southern regions of the YREB, have higher forest coverage than the average value of China. Subtropical forest productivity is next to that of the tropical rainforest globally speaking, and these forests are mainly distributed in China. Influenced by the East Asian monsoon, favorable hydrothermal conditions induce high CO_2_ uptake by subtropical forest in these areas (Yu et al., 2014) and induced the highest VC storage. The second largest natural forest of China is distributed in the western region of the YREB, and thus, this area also has a higher VC density than that of the plains of the eastern YREB (Figure 3a).

SOC density is decided by both bioproductivity and the decomposition rate of soil organic matter, and thus, it is mainly controlled by hydrothermal conditions and distribution of vegetation functional types. The mean annual temperature and precipitation during 1981‒2005 in the YREB were 14.2°C and 1,025 mm, respectively. The values range from 5 to 10℃ and 800 to 1,000 mm in alpine and subalpine areas of Western Sichuan. The subalpine and alpine area of the western region of the YREB represents the second largest natural forest of China. High bioproductivity is induced by diverse vegetation functional types which are attributable to the complex landform in this region (Tao et al., 2007), and therefore, this forest area is one of the most important carbon sink in China (Cao & Woodward, 1998b). Furthermore, alpine meadow and marshland are distributed in high‐altitude area of Western Sichuan. The area of marshland in Sichuan province is 117.59 × 10^4^ ha and accounts for 84.5% of total area of marshland in the YREB. For example, the largest wetland in Sichuan province, the Zoige wetland, distributed in the plateau with an altitude of more than 3,000 m. The mean annual temperature in the Zoige wetland is only 0‒2°C, much lower than the average value for the YREB. Relative cold conditions and adequate water supply prevail across this region due to high elevation, so the decomposition of soil organic matter is extraordinarily slow and soil organic matter is accumulated as peat (Gu et al., 2014; Wang et al., 2000). Cooler conditions and high bioproductivity are favorable to the accumulation of carbon in soil in this area, and thus, the western region of the YREB is also one of the highest SOC density areas in China (Tang et al., 2018; Wang et al., 2000; Yu et al., 2005).

### 3.2 The change of terrestrial carbon density under different climate change scenarios

Climate change increases the terrestrial carbon storage in the YREB under both scenarios (Table 1), and the increase of terrestrial carbon storage in the YREB by the end of 21st century will fall into the range of 3.98–6.25 Pg C relative to 1986‒2005 under different scenarios. Future climate change stimulates more carbon accumulation in vegetation, and the increases are 22.7 to 39.6% of total VC storage.

Different vegetation types show different responses to future climate change (Table 2). The absolute increases of terrestrial carbon density in forest are the highest under both scenarios; however, the terrestrial carbon density in shrubland has the highest relative increase and is projected to increase approximately by 9.6% under the RCP8.5 scenario compared with the mean value found for the time period between 1981 and 2005 (Table 2). The grassland has both the lowest absolute and lowest relative increase of carbon densities. In addition, all vegetation types have a higher increase of terrestrial carbon storage under the RCP8.5 scenario compared with those under the RCP4.5 scenario, because of the more aggressive warming and high CO_2_ emissions under the RCP8.5 scenario.

The responses of different vegetation types under two scenarios indicate the different sensitivity of vegetation types to climate change and CO_2_ fertilization. Although global terrestrial NPP increased due to climate change, the response of ecosystem productivity varied spatially because temperature, radiation, and water impose varying limitation on vegetation in different parts of the world (Nemani et al., 2003). Franklin et al. (2016) reported that many researches focused on the responses of vegetation changes on multiple scales, such as species, communities, landscapes, and global scales. They found that warming stimulated the growth and productivity at areas of high latitude, which are temperature limited, and enhanced water deficit resulted in decreased carbon flux and carbon storage especially in areas that were water limited (Franklin et al., 2016; Wu et al., 2011). Increasing carbon sinks in the northern hemisphere temperate latitudes are owing to the warming and the CO_2_ “fertilization effect,” with effects on growth being greatest for woody plants (Franklin et al., 2016). However, there is still lack of research on the sensitivity of productivity and carbon sequestration of different plant functional types to climate change, and thus, it is still a challenge to understand the mechanisms that influence the sensitivity of different plant functional types to climate change.

**FIGURE 4:**
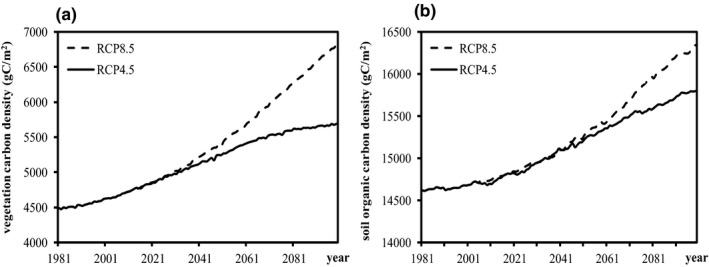
The increase of VC (a) and SOC density (b) during 1981‒2099 under different scenarios

Obviously, the magnitude of both VC and SOC density increase is much higher in future scenarios than that over the past 25 years, which is shown to be much higher under the RCP8.5 scenario than that under the RCP4.5 scenario (Figure 4). Under the RCP4.5 scenario, the increasing trend of VC density is reduced after the 2060s, probably because the temperature tends to be stabilized and greenhouse gas emissions peak around 2040; however, the increased rates of VC and SOC density both accelerate after the 2050s under the RCP8.5 scenarios (Figure 4a, b). Increased temperature rising and elevated atmospheric CO_2_ concentration contribute to the projected higher increase of terrestrial carbon storage in the future, especially under higher emission scenario.

Warming will stimulate plant growth and carbon uptake (Wu et al., 2011), as well as the respiration of plant and soil (Heimann & Reichstein, 2008). Meanwhile, the decrease in soil moisture contributes to the decrease in terrestrial carbon storage accompanied by warming and thus, the sensitivity of terrestrial carbon storage to climate change varies geographically (Peng et al., 2014). In the YREB, total VC density and SOC density both show sustainable growth influenced by past climate change and different future climate change scenarios (Figure 4). Temperature increases continuously from 1981 to 2099 and the increased rate is higher in the future compared to the period of 1981‒2005, and higher under the high‐emission RCP8.5 scenario. Precipitation shows large interannual variations and no significant trend at any time over the past 25 years, or in the future under the two RCP scenarios. The YREB is located in a subtropical humid zone, and annual precipitation ranges from 800mm to 1600mm. Benefited from the abundant rainfall in this region, warming shows positive effects on terrestrial carbon storage in the YREB and the decrease of soil moisture induced by the accompanying warming will not influence the vegetation growth and carbon uptake.

CMIP5 simulations suggested a consistent CO_2_ fertilization effect on terrestrial carbon storage (Peng et al., 2014). Atmospheric CO_2_ concentration will increase much higher under the RCP8.5 scenario than under the RCP4.5 scenario. The RCP4.5 scenario assumes that global annual greenhouse gas emission peak around 2040, then decline; therefore, the increase rate of VC density is reduced. In the RCP8.5 scenario, atmospheric CO_2_ concentration will continue to rise, and the increasing temperatures and elevated atmospheric CO_2_ concentration cause higher terrestrial carbon storage, which is increased under the RCP8.5 scenario compared to that under the RCP4.5 scenario.

The increase in VC density is higher than that of SOC density at all time points over the past 25 years and in the future under the two climate change scenarios. Previous research on Chinese forest also showed a higher increase rate of VC density than that of SOC density (Huang et al., 2016). The increase of VC density changes from 10% in the middle of the 21st century under the RCP4.5 scenario to 40% in the late 21st century under the RCP8.5 scenario; however, the increases of SOC density only ranged from 2% to 9%, even though many areas experienced a decrease in SOC density. This indicates that the warming stimulates productivity and biomass accumulation in the YREB; however, the effect of warming on carbon processes in soil is more complicated (Anderson, 1991; Heimann & Reichstein, 2008). Previous studies revealed that warming and increased precipitation both increased plant biomass and productivity and thus the carbon storage in vegetation (Wu et al., 2011). At the same time, warming would stimulate the respiration in plant and soil. Soil carbon stock is the equilibrium between litterfall carbon input and carbon release by decomposition of soil organic matter. Soil heterotrophic respiration exponentially increases with warming (Cox et al., 2000). When soil heterotrophic respiration exceeds the assimilation and carbon input stimulated by warming, a net carbon release from soil would be observed, which has been previously monitored by FLUXNET sites (Schwalm et al., 2010). Furthermore, the decline of soil carbon storage was found in some areas of the YREB, especially the wetland of alpine and subalpine region in the western YREB.

### 3.3 The spatial variations in response of terrestrial carbon density to future climate change

**FIGURE 5:**
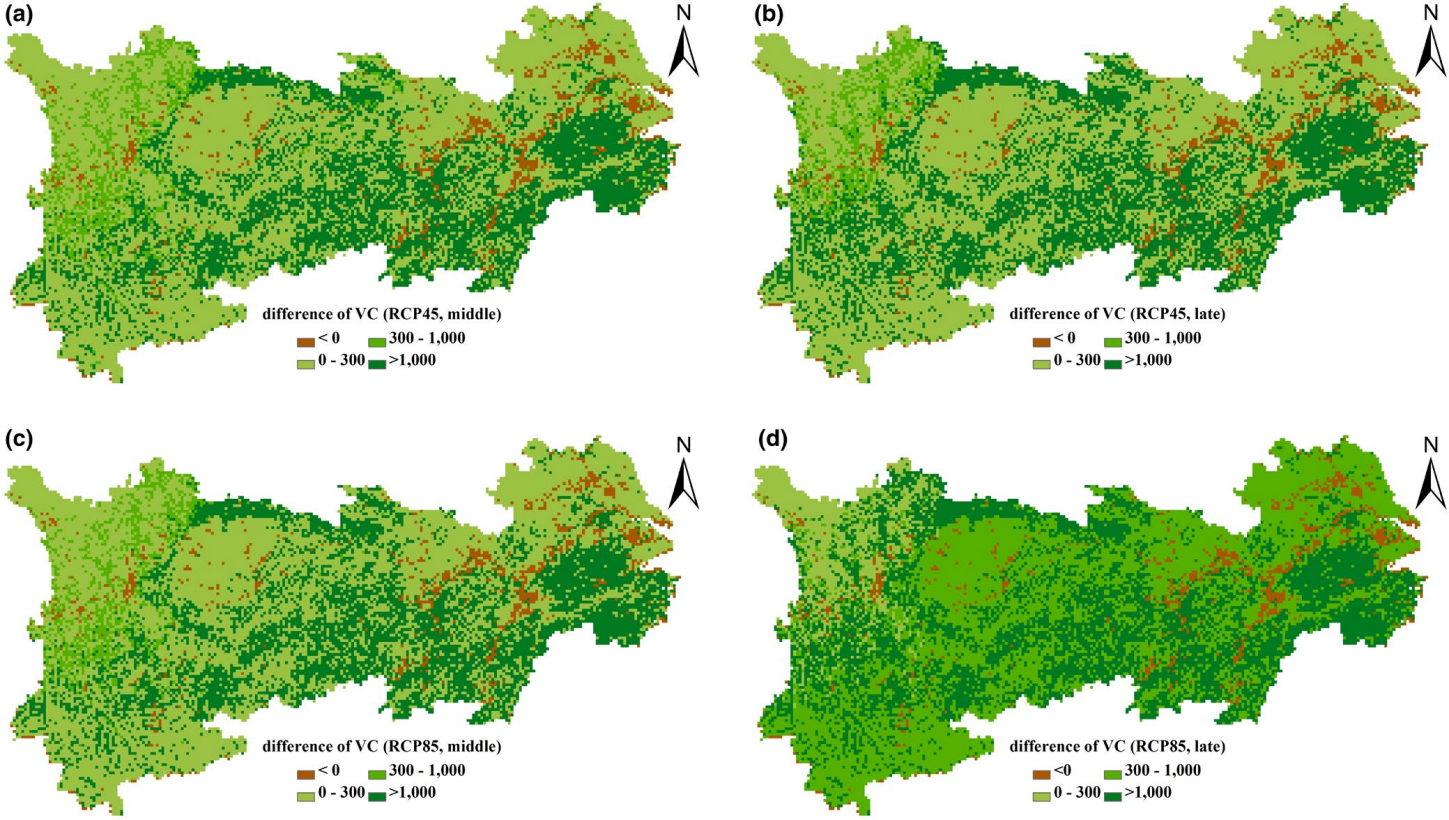
Changes in VC density, 2021‒2050, 2070‒2099 versus. 1986‒2005, as projected by the RCP4.5 (a, b) and the RCP8.5 scenarios (c, d). Unit: g C/m^2^

**FIGURE 6:**
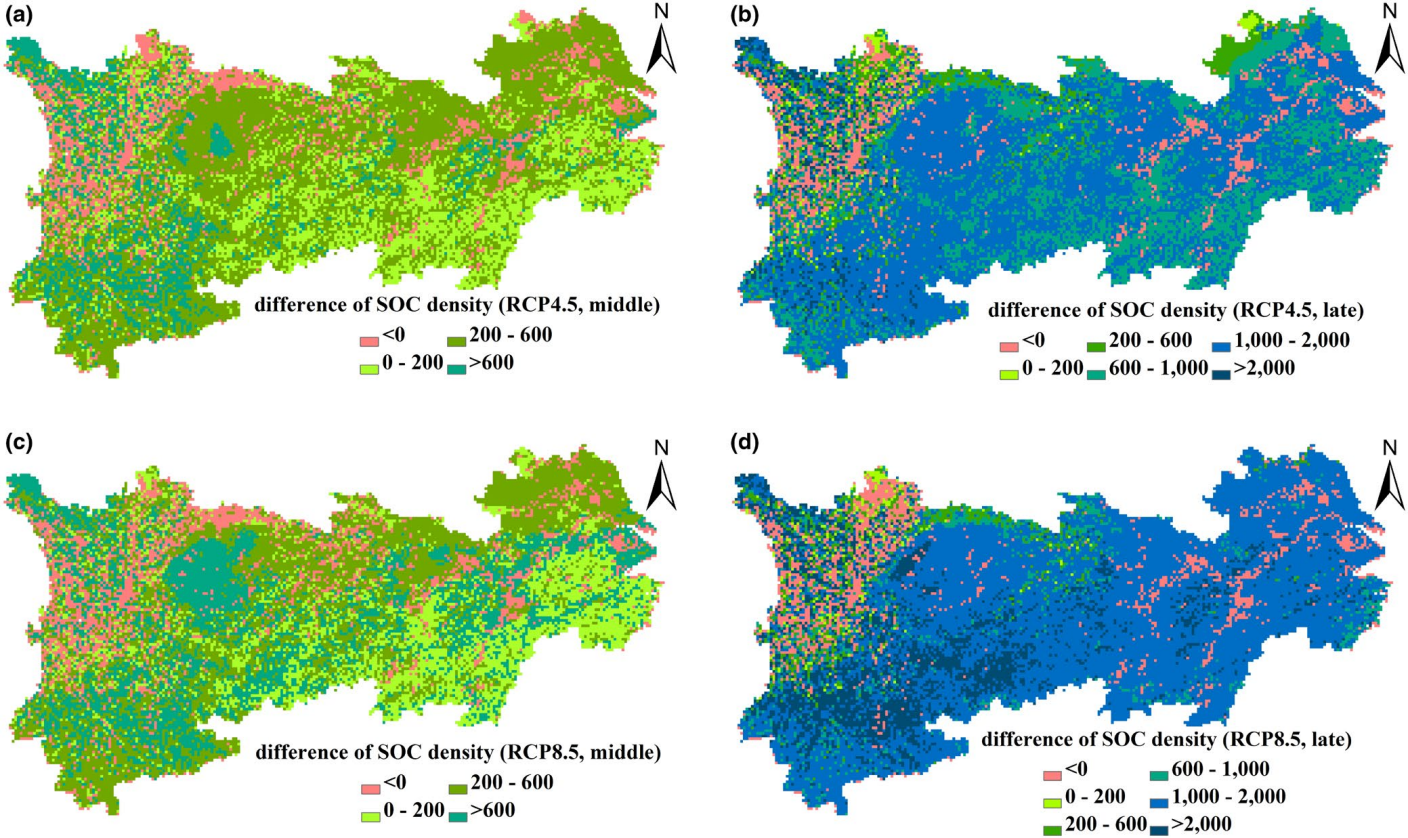
Changes in SOC density, 2021‒2050, 2070‒2099 versus. 1986‒2005, as projected by the RCP4.5 (a, b) and the RCP8.5 scenarios (c, d). Unit: g C/m^2^

Under the two RCP scenarios, a high increase in VC density appears in the southern YREB and surroundings of the Sichuan Basin (Figure 5). Only 8% of the area experienced decrease of SOC density in the middle of the 21st century, with a relatively higher increase of SOC density in central Yunnan province and some areas of Western Sichuan under the RCP4.5 scenario (Figure 6a, b). Under the RCP8.5 scenario, the decline of SOC density is further significant in the western region of the Sichuan province; however, the magnitude and distribution of SOC density increase also extends further than that under the RCP4.5 scenario (Figure 6c, d).

**FIGURE 7:**
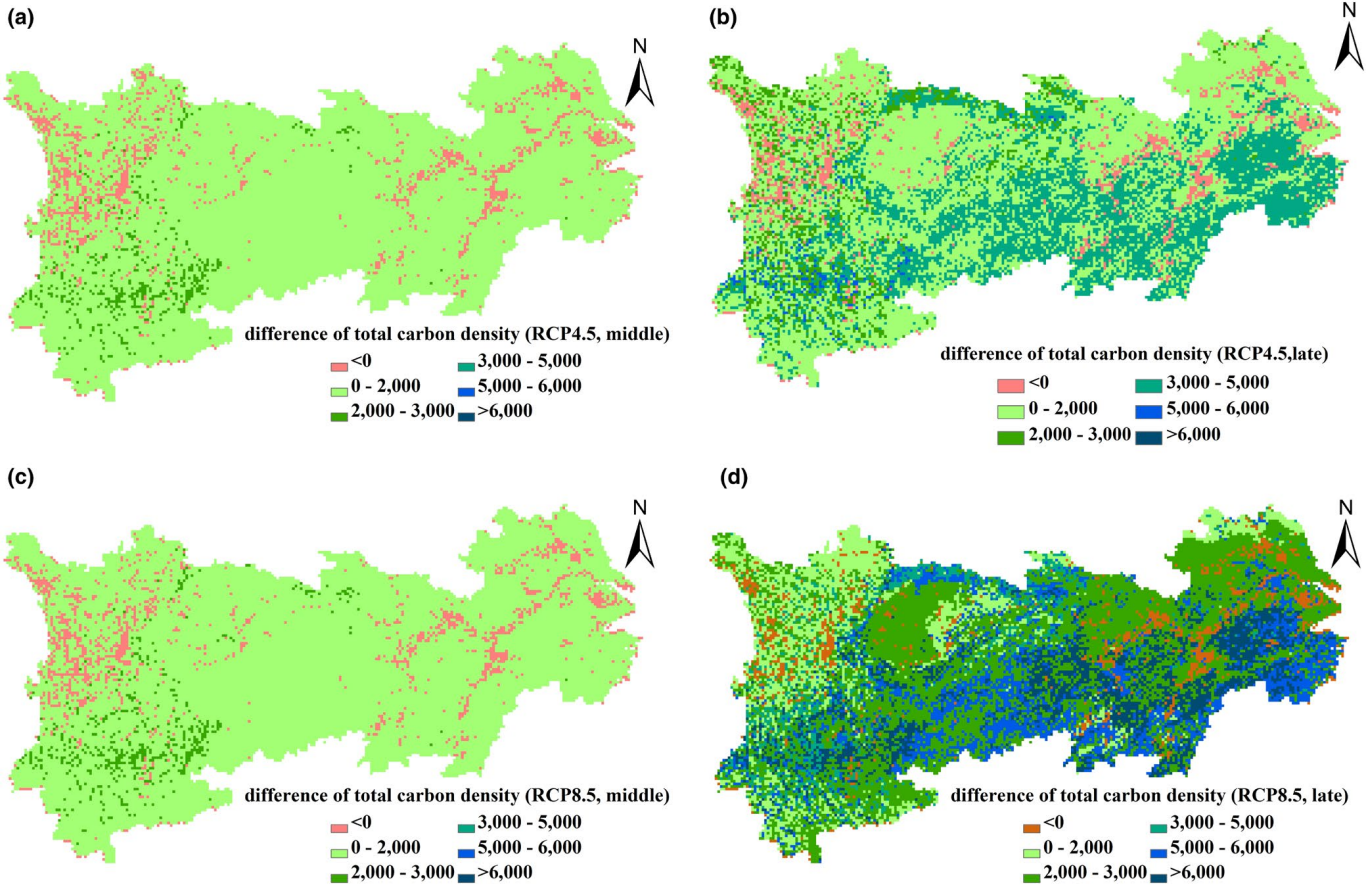
Changes in terrestrial carbon density, 2021‒2050, 2070‒2099 versus. 1986‒2005, as projected by the RCP4.5 (a, b) and the RCP8.5 scenarios (c, d). Unit: g C/m^2^

Determined by changes in both the VC and SOC density, an increase of terrestrial carbon density in the southern region and surrounding areas of the Sichuan Basin is higher than that in northern areas (Figure 7). Under the different RCP scenarios, the distribution patterns of VC, SOC, and terrestrial carbon density increase/decrease are similar between the middle and the late 21st century, but the increases in the late 21st century are substantially higher than those that occurred in the mid‐term (Figures 5, 6, 7).

Although the total vegetation, soil, and terrestrial carbon density in the entire region increased in the 21st century under different scenarios, the increase rates show large regional variation. The spatial variation of VC density change is largely depending on the sensitivity of different vegetation types to rising temperatures. The VC density increased in the entire region, stimulated by the increase in rising temperature and elevated atmospheric CO_2_ concentration, with the higher increase distributed in the southern YREB. Our results indicate forest has the highest increase rate. VC density of forests has a predicted increase rate of 25.73 g C/m^2^•yr and 50.70 g C/m^2^•yr under the RCP4.5 and the RCP8.5 scenarios, respectively, which are much higher than that of shrubland, grassland, and cropland. Forest is also considered as one of the three principal components of global terrestrial carbon sequestration (Lal, 2008). Meanwhile, the increase of VC density in subtropical evergreen broad‐leaved forest distributed in the southern region of the YREB is higher than that in evergreen coniferous forest distributed in the alpine and subalpine area of the western YREB (Figure 5a‐b). This indicates that the different forest types show different sensitivity to climate change.

The spatial variation of SOC density change is related to climate change and soil conditions. Our results show that SOC density decreased in some regions induced by continuous rising temperatures under both the RCP4.5 and the RCP8.5 scenarios, especially in the western region of the Sichuan province. More than 85% of evergreen needle‐leaf forest in the second largest natural forest area of China, located in Western Sichuan, experiences a decrease of SOC density. Compared with other regions in the YREB, it has a lower mean annual temperature but plentiful precipitation and the highest SOC density, which suggests that the SOC density in this area may be more sensitive to warming. Warming accompanied by an increase in precipitation stimulates the SOC efflux and SOC density decreases. In the future, efforts should become more concerned with the prevention of the net carbon emission of soil in Western Sichuan through ecosystem management.

## 4 THE UNCERTAINTY OF THE MODEL ESTIMATION AND FUTURE RESEARCH NEED

Many model simulations have suggested the positive effect of global warming and rising CO_2_ concentration on global terrestrial carbon storage in the future (Cox et al., 2013; Peng et al., 2014; Smith et al., 1992). The simulations on carbon storage of Chinese mature forests, based on the AVIM2 model and B2 climate change scenario during 1981‒2040, showed that the VC and SOC storage continued to increase (Huang et al., 2016). In addition, carbon sequestration in western forests of the United States has been projected to increase in the 21st century, although the potential of carbon storage could be limited by crown fires (Loudermilk et al., 2013). The simulations of different models showed large uncertainties on values and spatial variations of carbon storage change (Peng et al., 2014), because models varied in complexity and parameterization of different processes (Franklin et al., 2016). The uncertainty should be addressed by an intercomparison of multiple models.

The differences between the observations and simulations indicated that the accuracy of model simulation should be further improved. Many previous studies indicated that the sources of uncertainty in process‐based model simulations included model structure, input data, and selected parameters (Gu, Zhang, Huang, Tao, Guo, et al., 2017; Gu, Zhang, Huang, Tao, Li, et al., 2017; Ren et al., 2011; Zhang et al., 2012). Higher resolution input data, including land cover, climate, and soil parameters, can effectively reduce the uncertainty of model simulation in regions. In addition, more site‐observations are needed to calibrate the model to reduce the uncertainty of regional simulation results.

Changes to the terrestrial carbon cycle are influenced by multiple factors and their interactive effects (Ren et al., 2012; Tian et al., 2011). In this study, we analyzed the change of terrestrial carbon storage influenced by climate change and atmospheric CO_2_ concentration under different scenarios. In fact, various environmental factors changed dramatically and were frequently influenced by intensive human activities in the YREB. From 2000 to 2015, about 6.4 × 10^4^ km^2^ of ecosystems changed mostly to urbanization and ecological protection projects in the YREB (Kong et al., 2018). At the same time, forest cover has been increasing in the YREB (Yu et al., 2014). Over the past 20 years, the forest coverage in the YREB has increased 10.73%, and this increase in forest area contributed to 47.53% of the total increase of forested area in China, and in addition, the grain production increased 15.09% (https://data.stats.gov.cn/). Satellite data also showed that the YREB has led to the greening of China (Chen, Park, et al., 2019). All these environmental factors will undergo change and human disturbance will induce changes in vegetation structure in the YREB. Simulations made by the BEPS model indicated that a change in the structure of vegetation enhanced the global terrestrial carbon sink by 12.4% (Chen, Park, et al., 2019). The YREB is also one of the areas with the highest nitrogen deposition rate (Gu et al., 2015). A previous study showed that forest age, nitrogen deposition, and climate change explained more than 70% of CO_2_ uptake by subtropical forest in the East Asian Monsoon region (Yu et al., 2014). To reduce the uncertainty of the impacts of climate change on the regional carbon cycle, we should evaluate a multitude of drivers on terrestrial ecosystems not only climate change.

Under the high‐emission scenario, both VC and SOC storage showed a higher rate of increase influenced by rising temperature and CO_2_ fertilization. However, we should also note that SOC storage in some regions decreased and the underlying mechanisms which caused this should be discussed in depth. Furthermore, we should discuss whether more regions would show the decrease of SOC storage with the continuous rising temperature and atmospheric CO_2_ concentration and even the total terrestrial carbon storage of whole region would decrease in the future.

## 5 CONCLUSION

The carbon storage of the YREB plays an important role in the Chinese terrestrial carbon cycle. Both VC and SOC density in the YREB are higher than the mean values of Chinese terrestrial ecosystem. Although the area of the YREB only accounts for 21.4% of the entire country, the VC and SOC storages are up to 42.1% and 26.0% of China's total terrestrial ecosystem storages.

Climate change has already shown positive effects on carbon storage and is projected to have positive effects on the carbon storage in the YREB as well. Rising temperatures and elevated atmospheric CO_2_ concentrations stimulated the increase of carbon storage in the YREB. Vegetation, soil, and terrestrial carbon storage were found to increase continuously under both the RCP4.5 and the RCP8.5 scenario. High‐emission scenario has higher a rate of carbon storage increase. Forest has the highest rate of carbon storage increase, which contributes the most incrementally in carbon storage. However, rising temperatures will not always have positive effects as they cause the stimulation of the soil efflux and there may be different thresholds of climate that trigger the decrease of SOC storage according to different areas. For example, the SOC decrease with temperature rising in the western YREB, which is different from the response of SOC to temperature in other areas. All efforts in this study provide a vital ability and strategy to respond to future climate change for a well‐developed and important environmental region, including the YREB.

## CONFLICT OF INTEREST

The authors declare no conflict of interest.

## AUTHOR CONTRIBUTION

**Fengxue Gu:** Conceptualization (lead); Data curation (equal); Formal analysis (lead); Investigation (lead); Methodology (equal); Resources (equal); Software (lead); Supervision (equal); Validation (lead); Visualization (lead); Writing‐original draft (lead); Writing‐review & editing (lead). **Yuandong Zhang:** Conceptualization (equal); Data curation (equal); Formal analysis (equal); Investigation (equal); Supervision (equal); Writing‐review & editing (equal). **Mei Huang:** Conceptualization (equal); Data curation (equal); Funding acquisition (lead); Project administration (equal); Supervision (equal); Validation (equal); Writing‐original draft (equal); Writing‐review & editing (equal). **Li Yu:** Conceptualization (equal); Data curation (equal); Formal analysis (equal); Investigation (equal); Methodology (lead); Resources (lead); Software (equal); Writing‐original draft (equal); Writing‐review & editing (equal). **Huimin Gu:** Data curation (equal); Formal analysis (equal); Investigation (equal); Methodology (equal); Writing‐review & editing (equal). **Rui Guo:** Data curation (equal); Formal analysis (equal); Investigation (equal); Validation (equal); Writing‐review & editing (equal). **Li Zhang:** Conceptualization (equal); Data curation (equal); Formal analysis (equal); Investigation (equal); Methodology (equal); Resources (equal); Software (equal); Supervision (equal). **XiuLi Zhong:** Supervision (supporting); Writing‐review & editing (supporting). **Changrong Yan:** Project administration (supporting); Supervision (supporting); Writing‐review & editing (supporting).

## Data Availability

The authors declare that we will have the data publicly available at time of publication. The simulation data: Dryad, https://doi.org/10.5061/dryad.hhmgqnkd0.
